# Reply to Obegi, J.H. Distinguishing Prevention from Treatment in Suicide Prevention. Comment on “Turner et al. The Paradox of Suicide Prevention. *Int. J. Environ. Res. Public Health* 2022, *19*, 14983”

**DOI:** 10.3390/ijerph20095726

**Published:** 2023-05-05

**Authors:** Kathryn Turner, Anthony R. Pisani, Jerneja Sveticic, Nick O’Connor, Sabine Woerwag-Mehta, Kylie Burke, Nicolas J. C. Stapelberg

**Affiliations:** 1 Metro North Mental Health, Metro North Health, Brisbane, QLD 4029, Australia; 2 Center for the Study and Prevention of Suicide, University of Rochester Medical Center, Rochester, NY 14642, USA; 3 Gold Coast Primary Health Network, Robina, QLD 4226, Australia; 4 Clinical Excellence Commission, Sydney, NSW 2065, Australia; 5 Mental Health and Specialist Services, Gold Coast Hospital and Health Service, Gold Coast, QLD 4215, Australia; 6 Faculty of Health Sciences and Medicine, Bond University, Gold Coast, QLD 4215, Australia; 7 School of Psychology, The University of Queensland, Brisbane, QLD 4072, Australia; 8 Australian Research Council’s Centre of Excellence for Children and Families over the Life Course, Brisbane, QLD 4068, Australia

One of the aims of our paper “The Paradox of Suicide Prevention” is to promote greater discourse on suicide prevention, with a particular focus on the mental health models used for the identification of, and interventions with, individuals who come into contact with tertiary mental health services. Obegi (2023)’s comments [1] raise a number of helpful points regarding the terminology we used to describe aspects of suicide prevention in relation to how these terms are traditionally used in epidemiology and prevention science. Obegi’s questions provide an opportunity to reinforce and clarify the aims and message of our paper.

Obegi’s broadest objection is to our use of the term “suicide prevention” to encompass measures taken to support or intervene with individuals in tertiary care settings. For example, he states that safety planning or psychological interventions should not be discussed as steps to “prevent” suicide, because they are treatment, not prevention. While a technical distinction between prevention and treatment is important, it is not a reasonable objection in this context. The term “suicide prevention” is commonly used in government, healthcare, and research to refer to strategies across the prevention–treatment continuum. Three systematic reviews of suicide prevention strategies use “suicide prevention” to include strategies that traverse the prevention-to-recovery continuum [2,3,4]. The reviews detail strategies that range from public education health promotion initiatives to education campaigns for community gatekeepers such as schools, through to training in depression recognition and treatment for general practitioners and active follow-up of consumers of mental health services following discharge or suicidal crisis as exemplars of evidence-based “suicide prevention” strategies. We feel that it is impractical to describe it any other way; indeed, the abstract of Obegi’s own commentary refers to “clinical pathways to prevent suicide”, which makes perfect sense but is inconsistent with the narrow nomenclature he proposes.

In our paper, we acknowledge the importance of this overall epidemiological approach and suggest that funding and service models need to comprise a “balance” of both ends of the continuum: primary prevention and treatment and recovery [5].

Obegi argues that our use of “selected” and “indicated” within a hospital-involved population is not appropriate because “the populations already manifest suicidality”. This implies that it is suicidality that we are endeavouring to prevent, whereas our paper assumes that it is suicide that we are trying to prevent. There are a range of pre-conditions that can lead to suicide, including presentations with suicidality or distress, that can be a focus of preventative interventions.

He notes that preventative interventions, defined as including universal, selected, and indicated interventions, typically refer to interventions for individuals or groups prior to the onset of a disorder or issue. In considering this perspective, it is useful to consider how prevention science models have evolved over time. The World Health Organisation [6] defines disease prevention as “population-based and individual-based interventions” divided into three categories: primary (reducing likelihood of developing a disease or disorder), secondary (interrupting, preventing or minimising the progress of a disorder during early detection), and tertiary (halting progression of damage already done, aiming to minimize the burden of diseases and associated risk factors) prevention. This conceptualisation was first described as early as the 1950s. Gordon [7] extended this model during the 1980s by introducing the categories of universal (approaches that target the general community, such as via education or health promotion campaigns), selective (strategies that target a subgroup of a population that is at higher risk of experiencing a particular issue or disorder) and indicated (strategies that target individuals who have minimal but detectable signs or symptoms suggesting a disorder or higher degree of risk) prevention. The Institute of Medicine (IOM), some 20 years later, built on this work by defining a model that separates primary prevention from treatment and recovery (IOM, 1994). The IOM model distinguishes population-based primary prevention from treatment and recovery, such that once an individual is experiencing symptoms or issues that result in presentation to a hospital, they would be considered within the “treatment”. However, it is clear there is overlap in these conceptual models, and the terminology associated with each are still in use today.

In considering existing models of treatment and recovery within “The Paradox of Prevention”, we highlight that existing paradigms potentially miss a proportion of the community who do not meet existing criteria for receiving support within public mental health services based on current risk prediction models. The paper posits that we must think differently about for whom, when, how, and where we intervene with individuals experiencing distress that do not reach the level of acuity necessary for admission to a mental health service. With this in mind, the primary prevention approaches that distinguish between “selected” and “indicated” interventions within the broader community have potential utility for application within a clinical treatment and recovery setting. We have taken this broad population-based approach and extended it into a novel context. We argue that there is great utility in taking such an approach and applying it to the novel context of the tertiary mental health system.

More specifically, there is a section of the community that experiences distress and/or contextual factors that mean that they would not meet the criteria for “primary prevention”; nor, however, do they meet the criteria for “treatment”. To address this, we argue that there is a need for a paradigm shift in which the conceptualisation of suicide risk is moved from a focus on those identified as “high risk” (e.g., high lethality attempt alongside a diagnosis of serious mental illness) to include opportunities to intervene and prevent suicides that occur in those with lower risk (e.g., low lethality, no psychiatric diagnosis). The concepts of selective and indicated interventions have potential within this new paradigm to assist clinicians to broaden their mental model and consider the needs of the broader population of individuals who come into contact with mental health services. Clinicians are encouraged to take a “population-based” view of all people who present at hospital in distress or with suicidality. This new “mental model” provides significant motivation for clinicians to place consumers on a pathway, combining individualised engagement, assessment of modifiable risk factors, and a tailored, collaboratively developed care plan within a model of care that provides interventions for all hospital-presenting patients, thus offering a balanced approach that can consolidate the individual- and population-based suicide prevention focus. We argue that this paradigm shift has potential to maximise opportunities to reduce the likelihood of suicide for the hospital-presenting population. Furthermore, it also provides a driver for leaders of services and health system planners to consider the need for re-engineering services around this paradigm shift, and appropriately resourcing them.

## References

[1] Obegi J.H. (2023). Distinguishing Prevention from Treatment in Suicide Prevention. Comment on Turner et al. The Paradox of Suicide Prevention. *Int. J. Environ. Res. Public Health* 2022, *19*, 14983.. Int. J. Environ. Res. Public Health.

[2] Mann J.J., Apter A., Bertolote J., Beautrais A., Currier D., Haas A., Hegerl U., Lonnqvist J., Malone K., Marusic A. (2005). Suicide Prevention Strategies: A Systematic Review. JAMA.

[3] Zalsman G.M.D., Hawton K.F., Wasserman D.M.D., van Heeringen K.M.D., Arensman E.P., Sarchiapone M.M.D., Carli V.M.D., Höschl C.M.D., Barzilay R.M.D., Balazs J.M.D. (2016). Suicide prevention strategies revisited: 10-year systematic review. Lancet Psychiatry.

[4] Mann J.J., Michel C.A., Auerbach R.P. (2021). Improving Suicide Prevention Through Evidence-Based Strategies: A Systematic Review. Am. J. Psychiatry.

[5] Turner K., Pisani A.R., Sveticic J., O’Connor N., Woerwag-Mehta S., Burke K., Stapelberg N.J.C. (2022). The Paradox of Suicide Prevention. Int. J. Environ. Res. Public Health.

[6] World Health Organization (WHO) (2004). Global Forum on Chronic Disease Prevention and Control (4th: 2004: Ottawa, Canada).

[7] Gordon R.S. (1983). An Operational Classification of Disease Prevention. Public Health Rep..

